# Supplementary material for: Chimeric Antigen Receptor (CAR) T Cells Releasing Soluble SLAMF6 Isoform 2 Gain Superior Anti-Cancer Cell Functionality in an Auto-Stimulatory Fashion
Source: Cells. 2025 Jun 14;14(12):901. doi: 10.3390/cells14120901 (PMC12191382; doi:10.3390/cells14120901)
Supplement: Supplementary file 1 [file cells-14-00901-s001.zip › cells-3654786-supplementary.pdf]

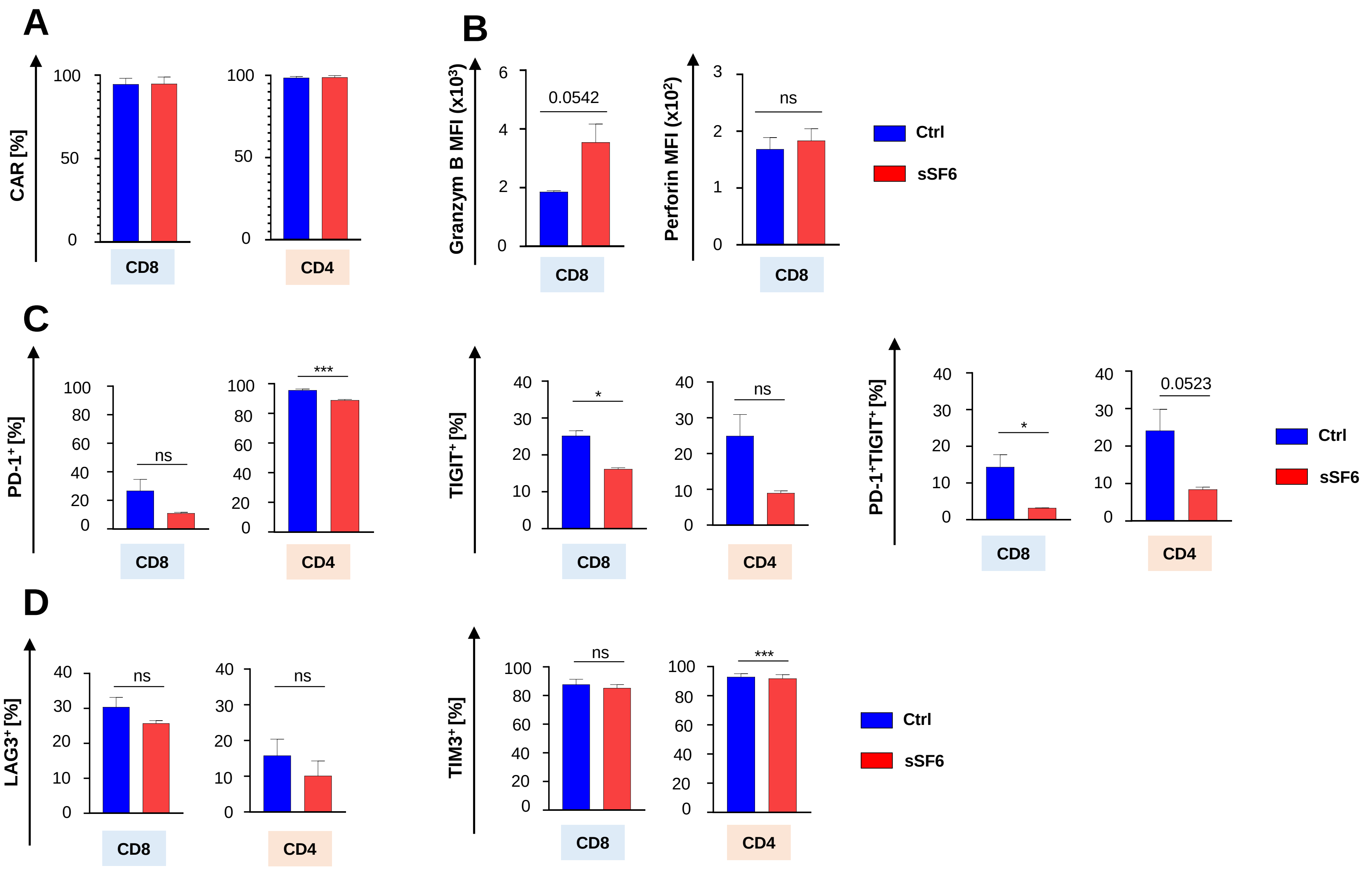

Supplemental Figure 1: Phenotypic analysis of CAR T cells during repetitive antigen stimulation. CAR T cells (CEA-28ζ-sSF6 = sSF6; CEA-28ζ-GFP = Ctrl) underwent three rounds (R1-R3) of antigen-stimulation with BxPC-3 cells. At the end of round three, CART cells were stained for CAR expression **(A)**, expression of granzyme B and perforin **(B)**, frequency of PD-1 positive, TIGIT-positive, and PD-1 positive TIGIT positive CD4+ and CD8+ CAR T cells **(C)**, and frequency of LAG3 positive and TIM3-positive CD4+ and CD8+ CAR T cells **(D)**. **(A-D)** Data represent means ± SEM of three donors, p values were calculated by Student's t-test in conjunction with Welch's correction, ns indicates not significant, * $p \leq 0.05$, and *** $p \leq 0.001$.

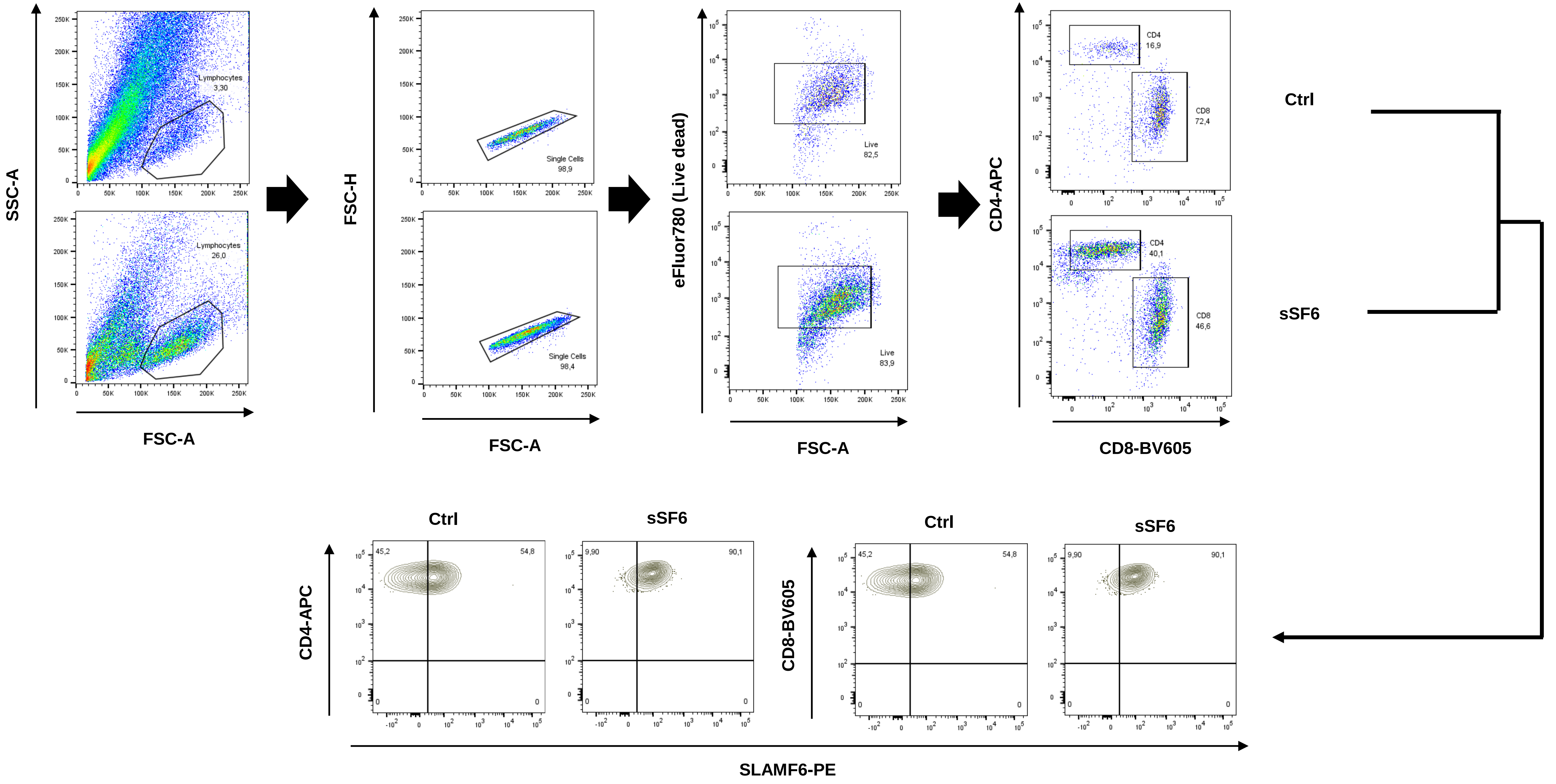

Supplemental Figure 2: Gating strategy for staining SLAMF6 on CAR T cells after three rounds of repetitive stimulation. First gate on lymphocytes, followed by exclusion of doublets and dead cells (live dead marker eFluor 708 in APC-Cy7 channel). Next, separation of CD8- and CD4-positive cells followed by staining for SLAMF6.

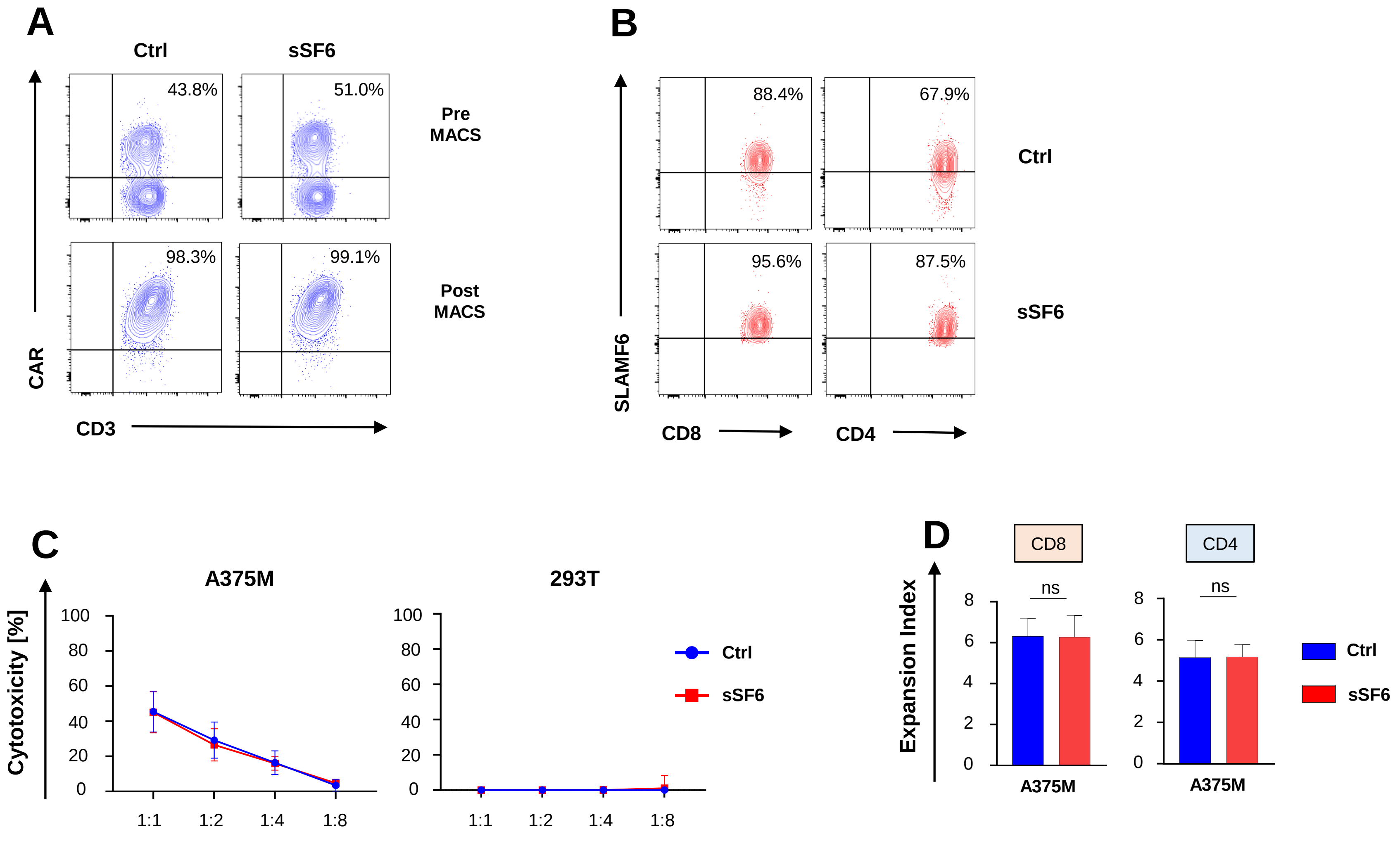

Supplemental Figure 3: Functionality of CSPG4-specific CAR T cells releasing soluble SLAMF6. (A) CAR expression on CAR T cells (CSPG4-28ζ-sSF6 = sSF6; CSPG4-28ζ-GFP = Ctrl) was determined prior (upper panels) and after (lower panels) magnetic cell separation (MACS). One representative donor out of three is depicted. **(B)** Baseline SLAMF6 expression on $CD8^+$ and $CD4^+$ CAR T cells (CSPG4-28ζ-sSF6 = sSF6; CSPG4-28ζ-GFP = Ctrl) at the start of in vitro assays. One representative donor out of three is depicted. **(C)** Cytolytic capacity of CAR T cells (CSPG4-28ζ-sSF6 = sSF6; CSPG4-28ζ-GFP = Ctrl) after a 24-hour co-culture with A375M cells and 293T cells at the indicated effector to target cell ratios. Data represent means ± SEM of three donors, p values were calculated by two-way ANOVA, ns indicates not significant. **(D)** Expansion index of $CD8^+$ and $CD4^+$ CAR T cells (CSPG4-28ζ-sSF6 = sSF6; CSPG4-28ζ-GFP = Ctrl) labeled with Cell Proliferation Dye eFluor® 450 and activated with A375M cells for five days. Data represent means ± SEM of three donors, p values were calculated by Student's t-test in conjunction with Welch's correction, ns indicates not significant.
